# Application of ventriculoscopy in granulomatous amoebic encephalitis: a case report in China and literature review

**DOI:** 10.3389/fmed.2024.1431225

**Published:** 2024-11-20

**Authors:** Zequan Zheng, Fajun Chen, Lulu Qin, Aili Lu, Haoyou Xu, Min Zhao, Yuanqi Zhao

**Affiliations:** ^1^Department of Neurology, The Second Affiliated Hospital of Guangzhou University of Traditional Chinese Medicine, Guangzhou, China; ^2^Guangzhou University of Traditional Chinese Medicine, Guangzhou, China; ^3^The Second Clinical Medical College, Guangzhou University of Chinese Medicine, Guangzhou, China

**Keywords:** amoeba, *Balamuthia mandrillaris*, meningoencephalitis, granulomatous amoebic encephalitis, ventriculoscope

## Abstract

*Balamuthia mandrillaris* is a kind of brain-eating amoeba leading to granulomatous amoebic encephalitis (GAE) with a high mortality rate and limited effective treatment options. Its underlying pathological mechanisms are still poorly understood. Here, we presented a 58-year-old man with *Balamuthia mandrillaris* meningoencephalitis, who died 33 days after onset. In this unique case, we introduced the use of ventriculoscopy in the treatment of *B. mandrillaris* meningoencephalitis. Through ventriculoscopy, we observed that the inflammatory response triggered by the disease was diffuse and extensive throughout the ventricles and meninges, not limited to the region of parasite invasion. Furthermore, under the precise guidance of ventriculoscopy, we implemented drug lavage therapy and intraventricular drainage, which, to a certain extent, managed to mitigate the progression of hydrocephalus and intracranial hypertension. Despite the unfortunate outcome of the patient’s death due to brainstem hemorrhage, we reiterate the crucial role that surgical intervention plays in both diagnosing and managing amoebic encephalitis. This underscores the need for continued research and innovation in surgical techniques to enhance patient outcomes and combat this challenging neurological condition.

## Introduction

*Balamuthia mandrillaris* is one genus of brain-eating amoeba first found in a mandrill baboon in the San Diego Wild Animal Park in 1989 (1). This parasite can cause a chronic and fatal infectious disease known as granulomatous amoebic encephalitis (GAE), with a mortality rate exceeding 95%. To date, only 11 survivors have been reported out of approximately 200 confirmed cases (2). The primary pathology is severe injury and extensive inflammatory edema in brain tissue (3), leading to fatal outcomes such as intracranial hypertension, hydrocephalus, and cerebral hemorrhage for patients. As detection technologies such as “next-generation” sequencing (NGS), polymerase chain reaction (PCR), and immunofluorescence develop, the diagnosis of amoebic encephalitis has become easier and faster. However, drugs often struggle to eradicate the pathogens and improve complications, necessitating neurosurgical interventions in most cases. In this study, we report a case of a 58-year-old Chinese man with meningoencephalitis caused by *B. mandrillaris,* who underwent ventriculoscopy and lateral external ventricular drainage (LEVD) to alleviate intracranial hypertension. Although the patient ultimately succumbed to cerebral hemorrhage, by analyzing the treatment process and comparing it with previous cases, we contend that surgical intervention holds significant value in the diagnosis and management of amoebic encephalitis.

## Case presentation

A previously healthy 58-year-old man was admitted to the hospital with a 5-day history of headache and fever (39.5°C). Prior to hospitalization, he had been assumed to have a cold and treated with antiviral medication. On the first day of admission, the patient’s physical examination revealed only an obvious stiff neck, with no skin lesions or other abnormal findings. He had been exposed to rain 1 day before the onset of symptoms but denied any contact with contaminated water or soil. We promptly conducted laboratory tests, which indicated that the intracranial pressure was within normal limits. However, the leukocyte count and protein quantification in the cerebrospinal fluid (CSF) were slightly elevated, and the initial cranial CT scan was normal. Based on these findings, we made a clinical diagnosis of suspected viral meningitis and initiated antiviral treatment with acyclovir. Nonetheless, the patient’s headache and fever failed to subside. Furthermore, he gradually developed symptoms of dizziness and blurred vision.

**Figure 1:**
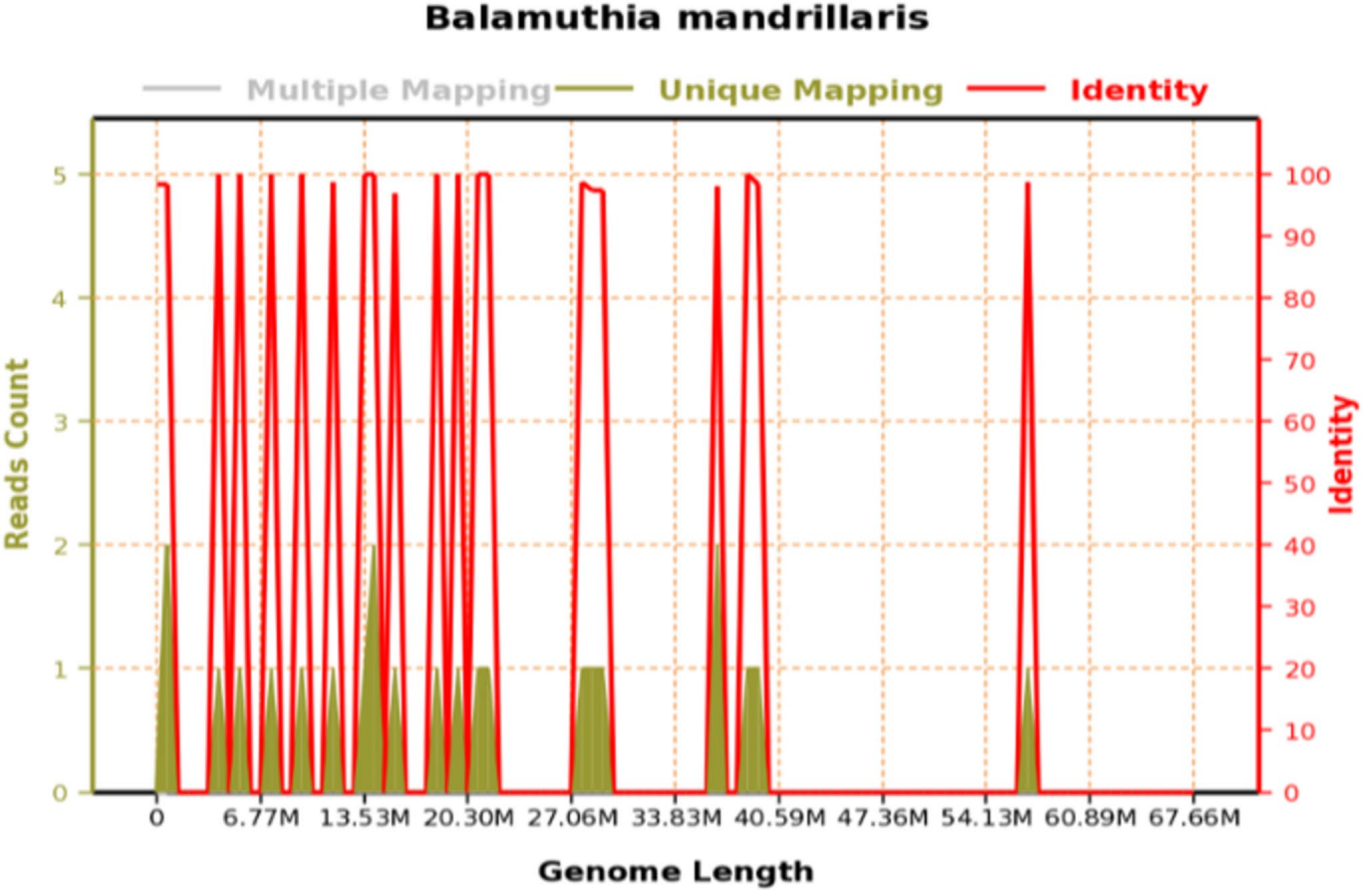
Genome coverage of *Balamuthia mandrillaris*: the total base number of the genome of this species is 67,656,513 (BP), the total length of the sequence coverage is 2,886 (BP), the coverage is 0.004266%, and the average depth is 1.00x.

**Figure 2:**
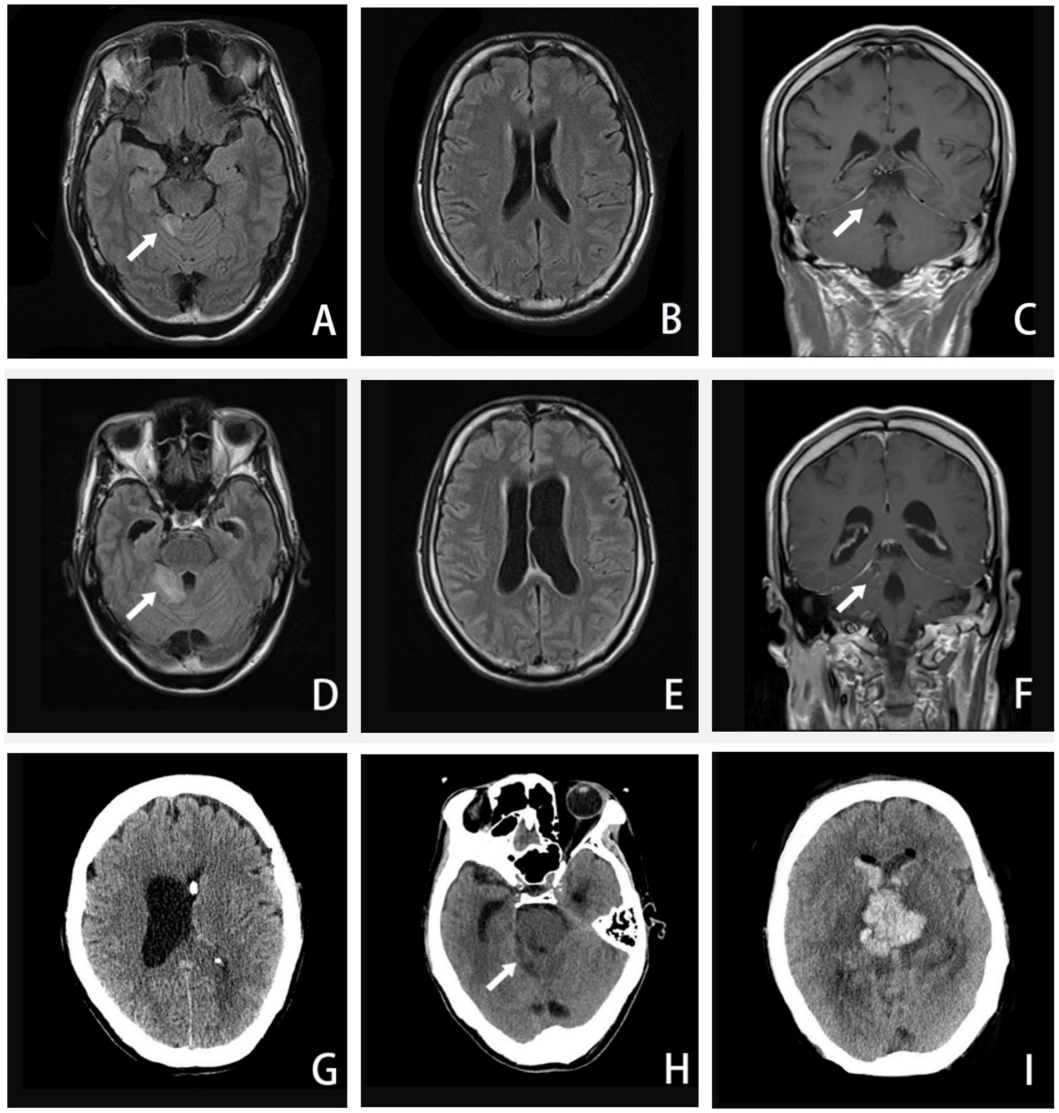
A–C, Axial FLAIR image and coronal T1-weighted enhanced image (16 September 2020) showing enhanced lesions on the cerebellar vermis and meninges. D–F, Axial FLAIR image and coronal T1-weighted enhanced image (24 September 2020) showing extended area on the cerebellar vermis lesion, along with ventricular expanded, especially the left side. G, CT image (27 September 2020) showing the left-side ventricular compressed by the expanded right-side. H–I, CT image (5 October 2020) showing the brainstem and ventricle with diffuse brain edema, inflammatory spread to the brainstem and the contralateral cerebellum, hemorrhage in the right-side of cerebellar vermis lesion. (The arrow in A, C, D, and F points to an enhanced lesion on the cerebellar vermis; the arrow in H points to brainstem and ventricle with brain edema).

Three days later, we were unexpectedly informed that the NGS result revealed an infection with an amoeba genus called *Balamuthia mandrillaris* (Figure 1). The second CSF test showed a higher CSF pressure and an increased leukocyte count compared to the first test, accompanied by a significant depletion of glucose. In addition, an enhanced brain MRI (Figures 2A–C) revealed extensive meningeal thickening and a lesion in the cerebellar vermis. Based on these findings, the patient was diagnosed with amoebic meningoencephalitis and treatment was initiated as follows: intravenous ceftriaxone 4 g/qd (one time a day), fluconazole 0.6 g/qd, and amphotericin B liposomes (started at an initial dose of 5 mg per day, gradually increasing to 40 mg per day). In addition, oral sulfamethoxazole 0.96 g/q8h (once every 8 h) and metronidazole 0.8 g/tid (three times a day) were prescribed. To alleviate intracranial inflammation and pressure, prednisone 20 mg/qd and mannitol 25 g/q12h (once every 12 h) were administered in combination.

Around the 14th day, the patient experienced a deterioration in consciousness and cognition, developing symptoms such as somnolence, memory dysfunction, disorientation in terms of time and place, nystagmus, worsening dizziness, and headache. A repeat MRI (Figures 2D–F) revealed an extended lesion in the cerebellar vermis and an enlargement of the left lateral ventricle, indicating obstructive hydrocephalus. With the consent of his family, our neurosurgeon performed a left-sided lateral external ventricular drain (LEVD) procedure on the patient. Following the surgery, he was transferred to the neurological intensive care unit (NICU) for further management.

**Figure 3:**
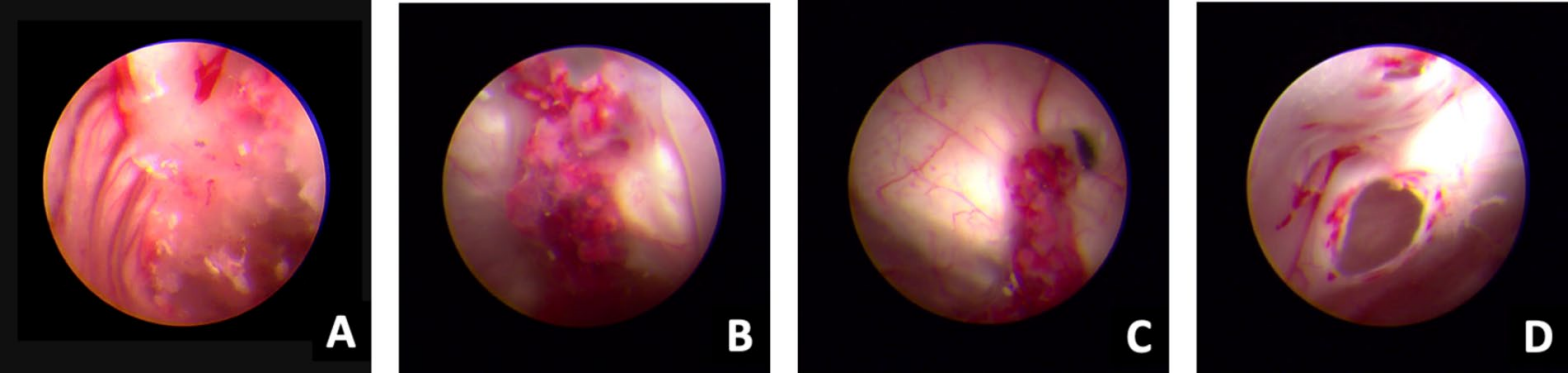
Ventriculoscope (27 September 2020). A–B, Vessels of the choroid plexus were congested and swollen, with partial bleeding, and the surface covered with jelly exudates and flocs. C, Ventricular foramen contracture under inflammatory state. D, Hole after EVT under ventriculoscope.

**Figure 4:**
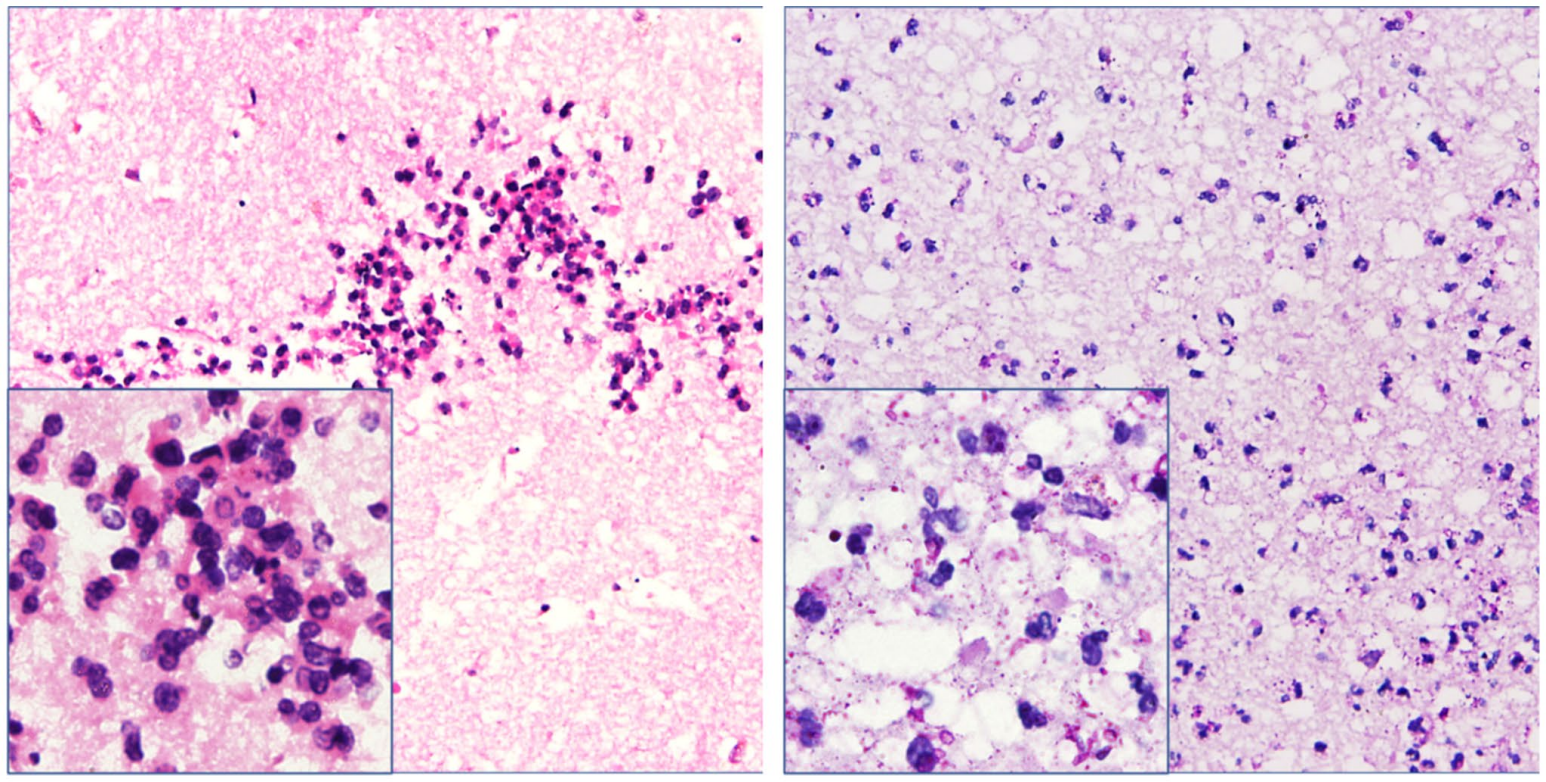
Necrotic tissue sections obtained from the operation showed inflammatory infiltration with neutrophils around the glial tissue and focal area. However, trophozoites and cysts were absent there (hematoxylin–eosin and periodic acid–Schiff staining, ×200 & × 400 mm).

Despite the interventions, the disease continued to progress. Three days after the initial left-sided LEVD, the patient required a right-sided LEVD, endoscopic third ventriculostomy (ETV), and intraventricular lavage due to obstruction of the drainage tube and worsening hydrocephalus evident on cranial CT (Figure 2G), which showed significant expansion of the right lateral ventricle. During the ETV procedure, a large amount of inflammatory exudate was observed covering the pia mater through the ventriculoscope (Figure 3). Histopathological examination of a small piece of brain tissue revealed inflammatory infiltration with neutrophils, but no trophozoites or cysts were present (Figure 4). These findings indicated an aggravating inflammatory response in the brain.

Twenty-four days after admission, the patient’s condition deteriorated significantly. In the morning, he experienced a seizure and rapidly progressed to a deep coma, accompanied by hypotension and loss of spontaneous breathing. His neurologic examination revealed mydriasis and the disappearance of brainstem reflexes. An urgent cranial CT examination showed a hemorrhage in the brainstem and ventricles, along with extensive brain edema (Figures 2H,I). Despite a week of life-sustaining therapy, the patient was eventually declared brain dead after evaluations using somatosensory evoked potential (SEP), electroencephalogram (EEG), and transcranial Doppler (TCD). Following this, his family decided to withdraw active treatment, and life support was withdrawn. The patient passed away 33 days after the onset of the disease (Table 1).

**Table 1:** Chronologic summary of the case history of the 58-year-old man with Balamuthia amoebic encephalitis.

Timeline	Presentation/status	Examination results	Laboratory finding	Treatment
Day 1 Outpatient department	Headache and fever			Oseltamivir, ibuprofen, and phoxim.
Day 5 Hospital admission		CT: normal	CSF pressure was 140mmHO2. CSF analysis revealed a slight rise in WBC (116 × 10^6/L), and protein was 1,037 mg/L.	Acyclovir (0.625 g ivd q8h)
Day 8	Headache, fever, dizziness, and blurred vision	MRI: an enhanced lesion was shown on the cerebellar vermis and meninges.	High-throughput sequencing test revealed the infection of *Balamuthia mandrillaris* whose sequence number was 23; CSF pressure was 220mmHO2 CSF-WBC (412 × 10^6/), glucose (2 mmol/), protein (1,185 mg/L)	Discontinued acyclovir. Started ceftriaxone (4 g ivd qd); sulfamethoxazole (0.96 g po q8h); metronidazole (0.8 g po tid); fluconazole (0.6 g ivd qd); amphotericin B liposomes (started at first dose of 5 mg ivd per day and gradually gave dosage to 40 mg per day). Combined with prednisone (20 mg po qd); mannitol (25 g ivd q12h).
Day 11	Headache， fever， dizziness, and nystagmus		NGS sequence number:3419	Discontinued ceftriaxone. Changed prednisone into dexamethasone (30 mg ivd qd); started moxifloxacin (400 mg ivd qd).
Day 14 Transfer to NICU	Somnolence， dysfunction of memory, orientation to time and place, headache, fever, dizziness, and nystagmus	MRI: the lesion on the cerebellar vermis extended, and the ventricles expanded, especially the left side.		Performed the left-side lateral external ventricular drain (LEVD).
Day 17	Light coma and fever	CT: the right-side ventricle expanded along with the left-side compression.		Performed the right-side lateral external ventricular drain (LEVD) in combination with pellucid septostomy and infusion of metronidazole.
Day 18				Started fluorocytosine (1.5 g po tid).
Day 20			CSF pressure was 220mmHO2 CSF-WBC (210 × 10^6/), glucose (4 mmol/), protein (9,421 mg/L)	
Day 23	Coma and fever			Started meropenem (2 g ivd q8h).
Day 24	Deep coma, fever, and seizure			Started vasoactive agent and mechanical ventilation.
Day 25	Mydriasis and disappearance of brainstem reflex	CT: hemorrhage in the right side of cerebellar vermis, brainstem, and ventricle with diffuse brain edema. Inflammatory spread to the brainstem and the contralateral cerebellum.		
Day 32	Diagnosed brain death	SEP: N13, P14, N18, and N20 wave disappeared; EEG: resting electroencephalogram; TCD: nail wave.		His family made a decision to stop active treatment.
Day 33	Declared clinical death			

## Discussion

Since the first reported case in 1990, *B. mandrillaris* has claimed over 200 human lives worldwide (3). Infection with *B. mandrillaris* is rare in our country. To date, there have been only 28 confirmed cases reported, of which 16 patients developed encephalitis (4). In contrast to the aggressive primary amoebic meningoencephalitis (PAM) caused by *Naegleria fowleri*, patients infected with *B. mandrillaris* experience a subacute or chronic course lasting weeks or even several months. This genus primarily causes GAE and skin lesions, rarely affecting other organs. The invasion can cause severe inflammatory damage to the brain, leading to fatal brain edema and intracranial hypertension (5). To date, there is still no clinical evidence or drug trial to demonstrate which antibiotic has a targeted therapeutic effect (6). Therefore, supportive treatments, such as hypothermia therapy, neurosurgery, and steroids, are often crucial measures to save and prolong patients’ lives (7). In our case, we pioneered the use of ventriculoscopy in the treatment of amoebic encephalitis. Through ventriculoscopy, we observed inflammatory changes on the meninges and treated the patient with intraventricular drainage and drug lavage. We aim to illustrate the advantages of ventriculoscopy in the treatment of this fatal intracranial parasitic infection through case analysis and literature review.

### Biological characteristics of *Balamuthia mandrillaris*

*Balamuthia mandrillaris* belongs to a genus of brain-eating amoebae that are most commonly found in soil, dust sources, and stagnant water (8, 9). The cyst walls are thick enough to provide protection for the amoebae against physical and chemical conditions in hostile environments. This barrier also aids in their resistance to antibiotics, leading to an increased likelihood of infection recurrence (8–11). It invades the body through skin lesions or the nasal mucosa and olfactory bulb, subsequently breaching the blood–brain barrier (BBB) and entering brain tissue, where it activates the intracranial immune response (12, 13). During this process, macrophages and neutrophils release a significant amount of pro-inflammatory factors, such as IL-1, IL-6, and TNF-*α*, accelerating the damage to the BBB and surrounding tissue (14). Following parasitic invasion, protozoan proteases and toxins can cause local damage, recruiting neutrophils and monocytes to infiltrate the area (15). This may ultimately result in a widespread inflammatory response, tissue edema, and hemorrhagic necrosis.

In our case, the patient initially presented with symptoms of meningitis and gradually developed symptoms of cerebellar injury, cortical dysfunction, and consciousness disorders. Through ventriculoscopy, we observed multiple meningeal injuries accompanied by a large amount of inflammatory exudates and floccules as well as some lesions with small hemorrhages. The vessels of the choroid plexus were congested and swollen, with partial bleeding, and the surface was covered with gelatinous exudates and flocs. Ventricular foramen contractures occurred due to the severe inflammation. It appears that vascular inflammation and necrotic hemorrhage are common in amoebic meningoencephalitis, which is also confirmed by hematoxylin and eosin staining and ventriculoscopy findings. We found varying degrees of inflammatory lesions in the meninges, brain parenchyma, and intracranial microvasculature in areas devoid of parasites. Ultimately, the patient succumbed to sudden intracranial hemorrhage caused by *Balamuthia mandrillaris*.

### Diagnosis of BAE

Clinicians are often unaware of amoebic meningoencephalitis at the early stage due to the lack of specific manifestations in its clinical presentation and neuroimaging (16–18). The results of laboratory tests are also suboptimal, as *Balamuthia mandrillaris* is difficult to detect in the CSF of infected patients, either visually by microscopy or through PCR. In a case series of 109 infected individuals, the CSF culture positivity rate was only 6% (6/109), and the majority of patients, nearly 88% (96/109), were diagnosed via brain biopsy through stereotactic aspiration or ventriculoscopy. However, NGS provides crucial clinical evidence for the early detection of pathogens. Currently, NGS has been capable of detecting multiple amoeba species, including *Acanthamoeba castellanii*, *Naegleria fowleri*, and *Balamuthia mandrillaris* (19, 20).

In our case, the patient was initially suspected of having a viral infection, presenting with only headache and fever. The mild abnormalities in the CSF and the normal CT image failed to provide any indication of a parasitic infection. However, NGS significantly contributed to the early diagnosis, confirming the patient’s infection with *Balamuthia mandrillaris* 3 days after admission. This prompt confirmation allowed us to immediately adjust the therapeutic regimen. Subsequently, we attempted to identify amoeba trophozoites through intraventricular biopsy, but unfortunately, pathological staining did not reveal typical manifestations. This may be attributed to the lesions being located in the infratentorial cerebellum rather than the ventricle. Nevertheless, surgical biopsy remains an indispensable tool in the diagnosis of amoebic encephalitis (6).

### Treatment for GAE

The current medical treatment of GAE is largely based on limited *in vitro* experimental data (21, 22) and successful case reports found in the literature (10, 23). As such, the prescriptions are often empirically formulated based on the few survival cases that have been documented. For instance, Vollmer reported a case series of 11 survivors with documented antimicrobial therapy regimens, in which azoles were the most commonly used drugs (9 out of 10 survivors), including 6 treated with fluconazole and 4 with itraconazole (2). In 2017, the United States Centers for Disease Control and Prevention (CDC) published a recommended medication regimen for GAE (24). In our case, a combination of three azoles along with amphotericin B and sulfamethoxazole was used to treat the amoeba infection after the diagnosis was confirmed. However, the treatment appeared to be ineffective based on the worsening clinical symptoms and the patient’s eventual demise. The primary reason for this could be that these drugs do not effectively penetrate the blood–brain barrier and are ineffective against the cyst wall of *Balamuthia mandrillaris*, which is known to be a significant challenge in treating this infection.

Brain edema, meningitis, and intracranial hypertension caused by GAE are often life-threatening and constitute the primary causes of mortality in these cases. In situations where there is progressive hydrocephalus and intracranial hypertension, steroids and dehydration drugs may not be sufficient to reduce inflammation and improve symptoms. In such instances, neurosurgery becomes more crucial than medication and is often a vital measure for saving and prolonging lives. Early-stage neurosurgery is reported to be important in both the diagnosis and therapy of GAE (25). LEVD is commonly used to treat hydrocephalus, but in parasitic infections, there is often a high failure rate of drainage, leading to poor prognosis. However, with the advancements in endoscopic neurosurgery, ETV has significantly reduced the failure probability of LEVD and effectively controls intracranial pressure (26). In some cases, the ventriculoscopic approach to infected focus resection has been reported as an effective technique with unique advantages, improving the total removal rate of intraventricular parasitic infections (27). Ventriculoscopy also allows for visual assessment of the degree of inflammation within the ventricles during the treatment of intracranial infection. During surgery, continuous irrigation with antibiotics can help kill pathogens, while also enabling the cleanup of local necrotic tissue and inflammatory secretions. These functions are crucial in controlling infections, reducing local inflammation, and ultimately improving patient outcomes (28).

In our case, the observation of diffuse intraventricular inflammation in patients infected with *B. mandrillaris* through ventriculoscopy highlights the importance of direct visualization in assessing the severity and extent of the infection. By performing irrigation on areas with obvious inflammation during surgery, we were able to target the source of the inflammation and potentially reduce its impact. In addition, taking tissue for biopsy during the operation was a crucial step in identifying the pathogen and guiding subsequent treatment. The initial use of LEVD to relieve intracranial hypertension and progressive hydrocephalus was appropriate as it helped to stabilize the patient’s condition in the early stages. However, the subsequent need for ETV and right-sided LEVD due to drainage failure underscores the challenges of managing these complex infections. Despite these challenges, the active surgical intervention was successful in controlling the intracranial pressure (ICP) within a safe range, preventing potentially devastating complications such as brain herniation caused by rapid increases in ICP. Although the patient’s ultimate outcome was tragic, it is important to acknowledge the positive role that ventriculostomy therapy and LEVD played in controlling intracranial hypertension and prolonging survival. In the face of parasitic infections with high mortality rates, such as those caused by *B. mandrillaris*, surgical supportive therapy remains a necessary component of treatment to save lives and prolong survival periods. This case serves as a reminder of the importance of early intervention, aggressive management, and the use of advanced surgical techniques in the treatment of these challenging infections.

## Conclusion

In summary, GAE is a life-threatening infectious disease characterized by a rising number of diagnosed cases and a significant mortality rate. In this study, we report a case of GAE diagnosed in Guangdong, China, utilizing NGS on the third day post-admission. Despite administering a range of recommended empirical antibiotics and surgical interventions, the patient succumbed to complications. This case underscores the following key points: (i) NGS emerges as a valuable diagnostic tool for early identification of suspected central nervous system infectious diseases; (ii) the significance of neuroendoscopy in the diagnosis and management of amebic meningoencephalitis merits emphasis, particularly for patients exhibiting ventricular infection, progressive hydrocephalus, intracranial hypertension, or requiring biopsy; (iii) in confronting this fatal infection, it is imperative to not only prioritize the pursuit of curative treatments but also emphasize the utilization of surgical procedures and various adjunctive therapies. This multifaceted approach can significantly extend patient survival and afford greater treatment opportunities.

## Data Availability

The original contributions presented in the study are included in the article/supplementary material, further inquiries can be directed to the corresponding author/s.

## References

[1] Visvesvara GS Martinez AJ Schuster FL Leitch GJ Wallace SV Sawyer TK. Leptomyxid ameba, a new agent of amebic meningoencephalitis in humans and animals. ASM J CD. (1990) 28:2750–6. doi: 10.1128/jcm.28.12.2750-2756.1990, PMID: 2280005 PMC268267

[2] Vollmer ME Glaser C. A Balamuthia survivor. JMMCR. (2016) 3:e005031. doi: 10.1099/jmmcr.0.005031, PMID: 28348755 PMC5330223

[3] Lorenzo-Morales J Cabello-Vílchez AM Martín-Navarro CM Martínez-Carretero E Piñero JE Valladares B. Is Balamuthia mandrillaris a public health concern worldwide? Trends Parasitol. (2013) 29:483–8. doi: 10.1016/j.pt.2013.07.009, PMID: 23988231

[4] Wang L Cheng W Li B Jian Z Qi X Sun D. Balamuthia mandrillaris infection in China: a retrospective report of 28 cases. Emerging Microbes Infect. (2020) 9:2348–57. doi: 10.1080/22221751.2020.1835447, PMID: 33048025 PMC7599003

[5] Visvesvara GS Moura H Schuster FL. Pathogenic and opportunistic free-living amoebae: Acanthamoeba spp., Balamuthia mandrillaris, Naegleria fowleri, and Sappinia diploidea. FEMS Immunol Med Microbiol. (2007) 50:1–26. doi: 10.1111/j.1574-695X.2007.00232.x, PMID: 17428307

[6] Cope JR Landa J Nethercut H Collier SA Glaser C Moser M. The epidemiology and clinical features of Balamuthia mandrillaris disease in the United States, 1974-2016. Clin Infect Dis. (2019) 68:1815–22. doi: 10.1093/cid/ciy813, PMID: 30239654 PMC7453664

[7] Orozco L Hanigan W Khan M Fratkin J Lee M. Neurosurgical intervention in the diagnosis and treatment of Balamuthia mandrillaris encephalitis. J Neurosurg. (2011) 115:636–40. doi: 10.3171/2011.4.JNS102057, PMID: 21619411

[8] Matin A Siddiqui R Jayasekera S Khan NA. Increasing importance of Balamuthia mandrillaris. Clin Microbiol Rev. (2008) 21:435–48. doi: 10.1128/CMR.00056-07, PMID: 18625680 PMC2493082

[9] Schuster FL Yagi S Gavali S Michelson D Raghavan R Blomquist I. Under the radar: balamuthia amebic encephalitis. Clin Infect Dis. (2009) 48:879–87. doi: 10.1086/597260, PMID: 19236272

[10] Bravo FG Seas C. Balamuthia mandrillaris amoebic encephalitis: an emerging parasitic infection. Curr Infect Dis Rep. (2012) 14:391–6. doi: 10.1007/s11908-012-0266-4 22729402

[11] Siddiqui R Ortega-Rivas A Khan NA. *Balamuthia mandrillaris* resistance to hostile conditions. J Med Microbiol. (2008) 57:428–31. doi: 10.1099/jmm.0.47694-0, PMID: 18349360

[12] Siddiqui R Khan NA. Balamuthia amoebic encephalitis: an emerging disease with fatal consequences. Microb Pathog. (2008) 44:89–97. doi: 10.1016/j.micpath.2007.06.008, PMID: 17913450

[13] Siddiqui R Khan NA. Biology and pathogenesis of Acanthamoeba. Parasit Vectors. (2012) 5:6. doi: 10.1186/1756-3305-5-6, PMID: 22229971 PMC3284432

[14] Siddiqui R Khan NA. Balamuthia mandrillaris: morphology, biology, and virulence. Tropical Parasitol. (2015) 5:15–22. doi: 10.4103/2229-5070.149888, PMID: 25709948 PMC4326988

[15] Baig AM Khan NA. Tackling infection owing to brain-eating amoeba. Acta Trop. (2015) 142:86–8. doi: 10.1016/j.actatropica.2014.11.004, PMID: 25445746

[16] Guarner J Bartlett J Shieh WJ Paddock CD Visvesvara GS Zaki SR. Histopathologic spectrum and immunohistochemical diagnosis of amebic meningoencephalitis. Modern Pathol. (2007) 20:1230–7. doi: 10.1038/modpathol.3800973, PMID: 17932496

[17] Michinaga S Koyama Y. Pathogenesis of brain edema and investigation into anti-edema drugs. Int J Mol Sci. (2015) 16:9949–75. doi: 10.3390/ijms16059949, PMID: 25941935 PMC4463627

[18] Singh P Kochhar R Vashishta RK Khandelwal N Prabhakar S Mohindra S. Amebic meningoencephalitis: spectrum of imaging findings. AJNR Am J Neuroradiol. (2006) 27:1217–21. PMID: 16775267 PMC8133936

[19] Chiu CY. Viral pathogen discovery. Curr Opin Microbiol. (2013) 16:468–78. doi: 10.1016/j.mib.2013.05.001, PMID: 23725672 PMC5964995

[20] Greninger AL Messacar K Dunnebacke T Naccache SN Federman S Bouquet J. Clinical metagenomic identification of Balamuthia mandrillaris encephalitis and assembly of the draft genome: the continuing case for reference genome sequencing. Genome Med. (2015) 7:113. doi: 10.1186/s13073-015-0235-2, PMID: 26620704 PMC4665321

[21] Lemke A Kiderlen AF Petri B Kayser O. Delivery of amphotericin B nanosuspensions to the brain and determination of activity against Balamuthia mandrillaris amebas. Nanomedicine. (2010) 6:597–603. doi: 10.1016/j.nano.2009.12.004, PMID: 20060497

[22] Schuster FL Guglielmo BJ Visvesvara GS. In-vitro activity of miltefosine and voriconazole on clinical isolates of free-living amebas: Balamuthia mandrillaris, Acanthamoeba spp., and *Naegleria fowleri*. J Eukaryot Microbiol. (2006) 53:121–6. doi: 10.1111/j.1550-7408.2005.00082.x, PMID: 16579814

[23] Siddiqui R Aqeel Y Khan NA. Killing the dead: chemotherapeutic strategies against free-living cyst-forming protists (Acanthamoeba sp. and Balamuthia mandrillaris). J Eukaryot Microbiol. (2013) 60:291–7. doi: 10.1111/jeu.12026, PMID: 23346945

[24] Laurie MT White CV Retallack H Wu W Moser MS Sakanari JA. Functional assessment of 2,177 U.S. and international drugs identifies the Quinoline Nitroxoline as a potent Amoebicidal agent against the pathogen Balamuthia mandrillaris. MBio. (2018) 9:5. doi: 10.1128/mBio.02051-18 PMC6212833 30377287

[25] Deol I Robledo L Meza A Visvesvara GS Andrews RJ. Encephalitis due to a free-living amoeba (Balamuthia mandrillaris): case report with literature review. Surg Neurol. (2000) 53:611–6. doi: 10.1016/S0090-3019(00)00232-9, PMID: 10940434

[26] Kaif M Husain M Ojha BK. Endoscopic Management of Intraventricular Neurocysticercosis. Turk Neurosurg. (2019) 29:59–65. doi: 10.5137/1019-5149.JTN.21389-17.3, PMID: 29465742

[27] Zhenye L Chuzhong L Xuyi Z Songbai G Peng Z Jiwei B. Ventriculoscopic approach for intraventricular Neurocysticercosis: a single neurosurgical Center's experience. World Neurosurg. (2017) 107:853–9. doi: 10.1016/j.wneu.2017.08.059, PMID: 28838876

[28] Guan F Huang H Ren ZY Wang ZY Fu JD Li YB. Neuroendoscopic evaluation and treatment for cerebral ventricular infection. Chin Med J. (2018) 131:2114–6. doi: 10.4103/0366-6999.239319, PMID: 30127223 PMC6111679

